# Insecticide resistance in malaria vectors in Kumasi, Ghana

**DOI:** 10.1186/s13071-016-1923-5

**Published:** 2016-12-07

**Authors:** Sandra Baffour-Awuah, Augustina A. Annan, Oumou Maiga-Ascofare, Soma Diloma Dieudonné, Priscilla Adjei-Kusi, Ellis Owusu-Dabo, Kwasi Obiri-Danso

**Affiliations:** 1 Kumasi Centre for Collaborative Research (KCCR), College of Health Sciences, Kumasi, Ghana; 2 Department of Theoretical and Applied Biology, College of Science, Kwame Nkrumah University of Science and Technology (KNUST), Kumasi, Ghana; 3 Bernhard-Nocht Institute for Tropical Medicine (BNITM), Hamburg, Germany; 4 Institut de Recherche en Sciences de la Santé (IRSS), Ouagadougou, Burkina Faso; 5 School of Public Health, College of Health Sciences, KNUST, Kumasi, Ghana

**Keywords:** Malaria, *Anopheles*, Resistance, Vector control, Urban area

## Abstract

**Background:**

There have been recent reports of surge in resistance to insecticides in pocketed areas in Ghana necessitating the need for information about local vector populations and their resistance to the insecticides approved by the World Health Organization (WHO). We therefore studied a population of malaria vectors from Kumasi in the Ashanti Region of Ghana and their resistance to currently used insecticides. We conducted susceptibility tests to the four major classes of insecticides by collecting larvae of anopheline mosquitoes from several communities in the region. Surviving adults from these larvae were then subjected to the WHO-approved susceptibility tests and characterization of knockdown resistance and acetylcholinesterase mutant genes.

**Results:**

Out of 619 *Anopheles* specimens sampled, 537 (87%) were identified as *Anopheles gambiae* (*sensu stricto*), which was also the species with the lowest knockdown resistance mutant gene, 61% (*P* = 0.017). Knockdown resistance mutant gene was as high as 91% in *An. coluzzii*. Mosquitoes collected showed susceptibility ranging from 98–100% to organophosphates, 38–56% to carbamates and 15–47% and 38–46% to pyrethroids and organochlorides, respectively. The knockdown resistance mutation frequency of *Anopheles gambiae* (*sensu lato*) mosquitoes that were exposed to both pyrethroids and organochlorides was 404 (65%). Acetylcholinesterase mutant gene was not found in this population of vectors.

**Conclusion:**

Our study shows that pyrethroids have the highest level of resistance in the population of mosquito vectors studied probably due to their frequent use, especially in impregnation of insecticide-treated nets and in insecticides used to control pests on irrigated vegetable farms. We recommend studies to monitor trends in the use of all insecticides and of pyrethroids in particular.

## Background

Malaria is transmitted by different *Anopheles* species and its occurrence and intensity is highly dependent on the region and the environment. *Anopheles gambiae* (*sensu stricto*) and *An. funestus* are the most common species of *Anopheles* in Ghana, followed by *An. coluzzii*, all found throughout the entire country. Two other species, *An. melas* and *An. rufipes*, are restricted to the southern and the northernmost parts of Ghana, respectively [1, 2].

Since indoor residual spraying (IRS) and long-lasting insecticidal nets (LLINs) are the main malaria vector control measures used in several sub-Saharan countries, the World Health Organization (WHO) has approved certain classes of insecticides to be used in their formulation namely, pyrethroids for both LLINs and IRS and organophosphates, carbamates and organochlorides for IRS only [3, 4].

Organophosphate and carbamate insecticides act irreversibly on inactivating the enzyme acetylcholinesterase which is essential for nerve function in mosquitoes [5]. The insecticidal effect of pyrethroids and DDT is due to insecticide binding to the sodium channel, modifying its gating properties, and keeping it open for a relatively longer period. Modifications in the sodium channel structure in the form of point mutations or substitutions as a result of single nucleotide polymorphisms confer insensitivity to DDT and pyrethroids. Although several chemicals are in use in urban areas, pyrethroids are the most widely selected and preferred class of insecticides for vector control because of their effectiveness, low toxicity to humans and high mosquito repellent properties [6]. The vectors of malaria are becoming increasingly resistant to many classes of insecticides in various parts of Ghana [7]. The upsurge in physiological insecticide resistance has multiple and interacting underlying mechanisms include metabolic detoxification, reduced insecticide infiltration and behavioural avoidance as well as cross-resistance [8].

The tendency for cross-resistance with respect to different classes of insecticides arises especially for groups sharing the same mode of action. The occurrence of cross-resistance will restrict the choice of alternative chemicals in situations where resistance has been detected. In view of the existence of cross-resistance, the WHO launched the Global Plan for Insecticide Resistance Management in Malaria Vectors (GPIRM) in May 2012, which is explicit on the need for closer monitoring of insecticide resistance, especially to pyrethroids [9].

There are several calls for the establishment of effective reporting mechanisms to ensure that information on species and insecticide resistance distribution are collected, assembled and fed back into the decision-making process [10, 11]. Vector control interventions will, therefore, be more responsive to the local situation leading to improved efficiency and effectiveness. As a result, there have been a number of published data from studies conducted mostly in small communities of Ghana [7, 12, 13]. Kumasi is a fast growing and urbanising city in Ghana and as expected there is high demand on food supply leading to exponential growth of irrigated agriculture in this urban setting. While the creation of irrigated farmlands serve as larval sites for the proliferation of *An. gambiae* (*s.l*.), there is a lack of data on the population of vectors as well as their resistance to the use of insecticides in an urban setting such as Kumasi. We therefore studied the use of major classes of insecticides recommended by the WHO in relation to different species of malaria vectors in this mosquito population.

## Methods

### Study area

The study was carried out in Kumasi Metropolis, Ghana. Kumasi lies in the southern belt of Ghana and covers 25,415 ha of the total land area of the country. Average annual rainfall is 625 m with peaks of 214.3 and 165.2 mm in June and September, respectively. This region lies in the forest zone where malaria transmission is intense and perennial. The level of disease transmission is high throughout the year because favourable environment exist [14].

The study site, Asokwa submetropolis in Kumasi Metropolis is the most populated of the five health submetropolis with a population of 495,375 (Kumasi Maps and Population, unpublished data). The submetropolis is made up of 26 communities and has the highest number of major irrigated vegetable farms in the metropolis. The vegetables cultivated on these irrigated farms include cabbage, green pepper, pepper, carrot, lettuce, spring onions, spinach, garden eggs, and cauliflower [15]. These fields represent highly productive larval sites and are found throughout Kumasi [16, 17].

### Mosquito sampling

*Anopheles* larvae and pupae were sampled from major open-spaced irrigated vegetable farms in the submetropolis and reared to adults in an insectary. The larvae were reared under standard conditions at 26–28 °C, 12:12 h photocycle and 70–80% relative humidity in the insectary. The adults were fed on 5% sugar solution soaked in cotton wool. Three to five day-old non-blood fed female adult *Anopheles* mosquitoes from each six major larval sites were then pooled and observed for an hour to ensure their fitness for insecticide susceptibility testing. *Anopheles* specimens were declared fit when they flew with all parts of their body intact. Any *Anopheles* that died, became immobile and/or lost any part of its appendages was declared unfit and discarded according to WHO requirement [18].

### Insecticide susceptibility assays

Insecticide susceptibility assays were performed on the wild larvae reared to adult in the laboratory using the standard WHO susceptibility test protocol and mortality rates calculated after 24 h [9]. A susceptible strain of *An. gambiae* (Kisumu) was used as reference strain for the bioassays. Insecticide-impregnated test papers with the WHO diagnostic dosages were supplied by the Universiti Sains Malaysia, Penang. Test papers were impregnated with pyrethroids (0.05% deltamethrin, 0.15% cyfluthrin, 0.05% lambda-cyhalothrin, and 0.75% permethrin); carbamates (0.1% propoxur and 0.1% bendiocarb); organophosphates (5.0% malathion and 0.25% pirimiphos-methyl) and organochlorides (4.0% dieldrin and 4.0% dichlorodiphenyltrichloroethane, DDT). For each insecticide, five tubes were prepared plus a tube for control. Twenty to twenty-five (20–25) randomly selected female *Anopheles* were used at 26–28 °C and 70–80% relative humidity.

The knockdown effect of insecticides on the mosquitoes were observed for every 5 min for the first 20 min and then every 10 min till the total time was an hour to obtain the knockdown effect (KD). Thereafter, mosquitoes were observed for 24 h with a piece of cotton soaked with sugar solution (5%) on the grille of the cork to feed the mosquitoes. The percentage of female *Anopheles* mosquitoes that died after the 24 h were recorded as the mortality rate for each insecticide all in conformity to WHO standards.

### Identification of *Anopheles* spp.

DNA was extracted from the legs and wings of dead and surviving *Anopheles* exposed to pyrethroids and organochlorides from WHO tube susceptibility test. The cetyl trimethyl ammonium bromide (CTAB) protocol was used [19]. Thereafter, downstream PCR for species identification of *An. coluzzii*, *An. gambiae* (*s.s*.) and *An. arabiensis* was performed as described previously [20].

### Resistance marker genotyping

To find out if target-site insensitivity were responsible for resistance in *Anopheles* after the WHO tube assay was performed, PCR genotyping of *kdr* and *ace-1* were carried out.

The allele specific PCR procedure for *kdr* genotyping was intended to detect the West African *kdr* allele, L1014F, using the protocol and primer sequence of Martinez-Torres et al. [21]. The L1014F was the only gene mutation analysed because it is the commonest in West Africa whereas the L1014S mutation is confined in eastern Africa [20]. Allele specific (AS) PCR (a conventional PCR) was chosen for *kdr* mutation detection in *Anopheles* mosquitoes although real time (RT) PCR is the most sensitive and specific assay to use. This PCR was however chosen on the basis of its relative lower cost and reports of small number of failed reactions and incorrect scores [22]. The primers AgD1 (5′-ATA GAT TCC CCG ACC ATG-3′) and AgD3 (5′-AAT TTG CAT TAC TTA CGA CA-3′) amplified the resistant allele yielding 195 bp fragments. The susceptible allele was assayed using primers AgD2 (5′-AGA CAA GGA TGA TGA ACC-3′) and AgD4 (5′-CTG TAG TGA TAG GAA ATT TA-3′), which amplified a 137 bp fragment. The primer set AgD1 and AgD2 amplified a common fragment of 293 bp for control. During amplification, denaturation was set at 94 °C for 3 min followed by annealing; 35 cycles (94 °C for 30 s, 55 °C for 30 s, 72 °C for 10 s). Extension was set at 72 °C for 5 min.

Similarly, PCR to detect *ace-1* G119S mutation as described by Weill et al. [23] was carried out with the primers, Ex3Agdir (5′-GAT CGT GGA CAC CGT GTT CG-3′) and Ex3Agrev (5′-AGG ATG GCC CGC TGG AAC AG-3′), which amplified a fragment of 541 bp. An initial denaturation for 3 min at 94 °C was run, followed by annealing, 35 cycles (94 °C for 30 s, 62 °C for 30 s, 72 °C for 20 s) and a final extension for 5 min at 72 °C. The PCR products (4 μl) were then digested with *Alu* I restriction enzyme according to the manufacturer instruction. The digestion was to produce a 403 bp fragment for susceptible homozygous mosquitoes (SS), and two fragments of 253 bp and 150 bp for homozygous resistant (RR). Heterozygous individuals (RS) show a combination of sensitive and homozygous resistant bands.

### Data management

All data were entered into Microsoft Excel 2010, double-checked to correct for errors and then exported to GraphPad Prism 5 for statistical analysis. In the case of a Gaussian distribution data, determined using the D’Agostino & Pearson omnibus normality test, one sample *t*-test and the parametric unpaired *t*-test were used while Mann–Whitney nonparametric test was used for data with a non-Gaussian distribution to estimate the differences in resistance between the different *Anopheles* species and insecticides and also against the Kisumu susceptible strain. Alpha level of 5% was accepted as statistically significant. The susceptibility levels of the mosquitoes were evaluated on the basis of the WHO criteria of test mortality [24].

## Results

### WHO insecticide susceptibility tube assay

All 1,197 *Anopheles gambiae* Kisumu strain were susceptible (98–100%) to the 10 tested insecticides which also presented with significant difference to the wild strain used in determining malaria vector insecticide resistance (*t*_(18)_ = 5.74, *P* < 0.0001). Bioassay results for these wild *Anopheles gambiae* (*s.l*.) from the Asokwa submetropolis was indicative of resistance to carbamates (38–56%), organochlorides (38–46%) and the pyrethroids (15–47%) but susceptible to the organophosphates (98–100%) (Table 1). There was a statistically significant difference in mortality of the *Anopheles gambiae* (*s.l*.) to each insecticide tested (*t*_(9)_ = 4.94, *P* = 0.0008). For each insecticide, the rate of mortality in the controls used were less than 5%, therefore no corrections using the Abbot’s formula were required.

**Table 1:** Resistance status of *Anopheles gambiae* (*s.l*.) strains collected at Asokwa submetropolis and Kisumu laboratory strains to the four main classes of insecticides recommended by WHO

Insecticide	Strain	*N*	Dead (*n*)	Survived (*n*)	Mortality (%)	Resistance (%)	95% CI	Susceptibility status
Organophosphates								
Malathion (5%)	Asokwa submetropolis	125	123	2	98	2	0.96–1.01	S
	Kisumu	124	124	0	100	0	–	S
Pirimiphos-methyl (0.25%)	Asokwa submetropolis	107	107	0	100	0	–	S
	Kisumu	125	125	0	100	0	–	S
Carbamates								
Propoxur (0.1%)	Asokwa submetropolis	119	67	52	56	44	0.47–0.65	R
	Kisumu	120	119	1	99	1	0.98–1.01	S
Bendiocarb (0.1%)	Asokwa submetropolis	99	38	61	38	62	0.29–0.48	R
	Kisumu	122	120	2	98	2	0.96–1.01	S
Pyrethoids								
Cyfluthrin (0.15%)	Asokwa submetropolis	109	34	75	31	69	0.22–0.40	R
	Kisumu	125	124	1	99	1	0.98–1.01	S
Deltamethrin (0.05%)	Asokwa submetropolis	102	48	54	47	53		R
	Kisumu	105	105	0	100	0	–	S
Lambda-cyhalothrin (0.05%)	Asokwa submetropolis	104	16	88	15	85	0.08–0.22	R
	Kisumu	107	106	1	99	1	0.97–1.01	S
Permethrin (0.75%)	Asokwa submetropolis	101	26	75	26	74	0.17–0.34	R
	Kisumu	124	124	0	100	0	–	S
Organochorides								
DDT (4%)	Asokwa submetropolis	108	41	67	38	62	0.29–0.47	R
	Kisumu	121	121	0	100	0	–	S
Dieldrin (4%)	Asokwa submetropolis	95	44	51	46	54	0.36–0.56	R
	Kisumu	124	123	1	99	1	0.98–1.01	S

*Abbreviations*: *N* total number, *R* resistant, *S* susceptible, *CI* confidence interval, *−* not determined

### Knockdown effect of insecticides

**Fig. 1:**
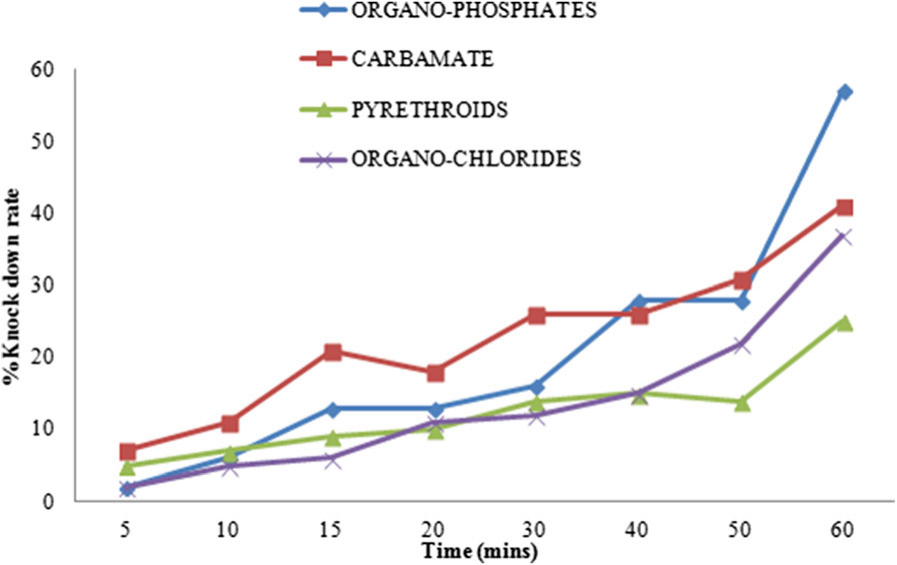
Knockdown rates of *Anopheles* mosquitoes from Asokwa submetropolis, Ghana, to the four major classes of insecticides approved by WHO. Rates within the first 60 min of exposure to the organochlorides, pyrethroids, carbamates and organophosphates are shown

As shown in Fig. 1, the organophosphates that also had the highest rate of mortality after 24 h had the highest knockdown (KD) (57%) after 60 min of exposure to the insecticide, followed by the carbamates (41% at 60 min). The pyrethroids had the least knockdown effect of 25% at 60 min.

### Molecular identification of *Anopheles gambiae* (*s.l*.) and characterization of resistance genes *kdr* and *ace-1*

Of the 1,069 female *Anopheles* mosquitoes used for the phenotypic detection of insecticide resistance, 619 were used for molecular *Anopheles* identification; of these, the majority (87%) were *An. gambiae* (*s.s*.) and the remaining 13% were *An. coluzzii*. Among the *An. coluzzii* and the *An. gambiae* (*s.s*.) identified, *kdr* mutation was detected at a frequency of 91 and 61%, respectively. This difference in *kdr* mutant gene detection between the different species was highly significant (*U*_(5)_ = 0, *Z * = 2.802, *P* = 0.002 (Table 2). Over a half of *An. gambiae* (*s.l*.) that survived the WHO tube assay test had *kdr* mutant genes but far lower proportions were recorded for those that died. All pyrethroid insecticides, of which permethrin was the highest, that *An. gambiae* (*s.l*.) survived during the tube assay test recorded with the highest *kdr* gene mutation (85–94%). DDT of the organochlorides reporting with the least *An. gambiae* (*s.l*.) *kdr* for those that survived however had the highest *kdr* mutant gene representation for those that died (Table 3). *Ace-1* mutation on the other hand was not detected among the female *Anopheles* mosquitoes subjected to organophosphates and carbamates.

**Table 2:** *kdr* resistance in *An. gambiae* (*s.s*.) and *An. coluzzii* from both dead and alive mosquitoes during the WHO tube assay test

	*kdr*-resistant *n* (%)	*kdr*-sensitive *n* (%)	Total *n* (%)
*An. gambiae* (*s.s*.)	329 (61)	208 (39)	537 (87)
*An. coluzzii*	75 (91)	7 (9)	82 (13)
Total	404 (65)	215 (35)	619 (100)

**Table 3:** *kdr* resistance of *An. gambiae* (*s.s*.) and *An. coluzzii* to organochlorides and pyrethroids during the WHO tube assay test segregated into dead and alive mosquitoes

Insecticide	*kdr*-resistant (alive) *n* (%)			*kdr*-resistant (dead) *n* (%)			*kdr*-sensitive *n* (%)		
	*An.*	*An.*	Total	*An.*	*An.*	Total	*An.*	*An.*	Total
	*gambiae*	*coluzzii*		*gambiae*	*coluzzii*		*gambiae*	*coluzzii*	
Organochorides									
DDT (4%)	53 (83)	13 (17)	66 (65)	19 (100)	0 (0)	19 (35)	21 (100)	2 (0)	23 (25)
Dieldrin (4%)	37 (65)	10 (35)	47 (83)	12 (100)	0 (0)	12 (17)	32 (60)	4 (40)	36 (10)
Pyrethoids									
Deltametrin (0.05%)	45 (88)	0 (12)	45 (85)	9 (82)	2 (18)	11 (15)	45 (100)	1 (0)	46 (24)
Permathrin (0.75%)	28 (67)	24 (33)	52 (94)	5 (100)	0 (0)	5 (6)	44 (100)	0 (0)	44 (25)
Cyfluthrin (0.15%)	49 (86)	26 (14)	75 (93)	9 (100)	0 (0)	9 (7)	25 (100)	0 (0)	25 (17)
Lambda-cyhalothrin (0.05%)	58 (86)	0 (14)	58 (93)	5 (100)	0 (0)	5 (7)	41 (100)	0 (0)	41 (17)

## Discussion

In Kumasi, *An. gambiae* (*s.l*.) was seen to be widespread with majority (87%) being *An. gambiae* (*s.s*.), in agreement with similar studies in the country [7, 25, 26]. The lower proportion of *An. coluzzii* was not surprising since this species is mostly linked to bigger and more permanent larval sites found in rice paddy fields, whereas *An. gambiae* (*s.s*.) are normally sampled from temporary, rain-filled larval sites in vegetable growing areas [27–29]. Klinkinberg et al. [28] who worked on a comparable urban setting in the country also recorded similar *An. gambiae* (*s.s*.) and *An. coluzzii* proportions among adult mosquito population. Interestingly, Afrane et al. [17] found no *An. coluzzii* while sampling in Kumasi over a decade ago. Bearing in mind that the sampling carried out by Afrane et al. was at the same location as this study, it depicts a gradual emergence of *An. coluzzii* population over the years in the said area. This probably is due to the advent of copious dug out wells for all year round irrigation purposes which are thus more permanent hence an attractant for this specie complex. This said, subsequent studies need to be carried out to affirm or otherwise this observation.

Only *An. coluzzii* contributed to malaria transmission with a high level of anthropomorphism and EIR in a study carried out in Ghana [30]. This means that the appearance of *An. coluzzii* has a significant implication for malaria transmission. This baseline results however require substantiation on a larger sampling scale and during different and extended periods in the year so as to utilize such localized malaria entomological data, which relates to the behaviour of the vector. This will definitely assist in the planning and implementation of more focused malaria vector control programmes. Especially in the event of such high Leu-Phe *kdr* mutation, which is the main mechanism of insecticide resistance in malaria vectors to pyrethroids in West Africa noted among this population of *An. coluzzii* (91%)*.* This elevated level of *kdr* mutation in *An. coluzzii* has been in a steady rise since the early 2000’s after which there was a 15-fold increase by 2011 [1, 31]. Before the year 2000, Chandre et al. [32] associated *kdr* mutation with *An. gambiae* (*s.s*.) only but not *An. coluzzii*. The reason for this possible crossover in *kdr* frequencies to *An. coluzzii* is conceivably through introgression from the *An. gambiae* (*s.s*.) [33]. However, further work needs to be carried out to ascertain whether this introgression incidence is what is actually pertaining in the setting of this study.

While *ace-1* gene mutation was absent contrary to other reports in some sub-Saharan countries [34, 35], *kdr* gene mutation was detected at 65% in *An. gambiae* (*s.l*.). The degree of *kdr* mutation observed was as high as reported in other parts of Ghana and West Africa [31, 36, 37].

The development of phenotypic and genotypic resistance to WHO-approved insecticides by *Anopheles* mosquitoes has been linked to agriculture [4, 27]. It is estimated that 87% vegetable farmers in Ghana principally rely on insecticides for pest control [38, 39] due to their effectiveness and rapidity in curative action [40]. However, during insecticide application, chemicals seep into larval sites. Mosquito larvae present are thus continuously exposed. Exposure to these insecticides could lead to an increase in resistance in mosquitoes [5]. Alarmingly, pyrethroids, which are used in ITNs, are the most widely used insecticides by farmers because of their photostability, enhanced insecticidal activity and relatively low toxicity as compared to the others [41]. Unfortunately, application of insecticides in agriculture is not regulated in Ghana. They are therefore used extensively, indiscriminately and even in overdose [25], explaining the pronounced resistance effect on vector mosquitoes as seen here. As a matter of urgency, application of insecticides by farmers should be well monitored by employing locals who will patrol and educate farmers on alternative pest control tools.

On a large scale, IRS and LLINs, the main malaria vector control tools in Ghana have significant variations with respect to regional coverage. LLINs are the focal malaria control tool in Ghana, increasing from 2.2% in 2003 to 48.9% in 2011 [42–44]. All of the ten regions in the country have been covered with the ‘door to door’ campaign that was implemented from 2010 to 2012 having an impressive coverage of 96.7% [44]. IRS however was designed initially to focus on 40 selected districts with two rounds of application based on the burden and technical practicability. As at the end of 2013, the plan was modified due to Global Fund allocation requiring a reduction of the districts to 12 of which all have been covered. The main facilitators of IRS in Ghana are AngloGold Ashanti Malaria Control (AGAMal) and the United States Agency for International Development- President’s Malaria Initiative (USAID-PMI) [45, 46]. In addition to IRS and LLINs, there are also household use of insecticide consumer products such as aerosol, mosquito coils, liquid vaporizers and repellents [47, 48]. In all these, the most common chemical used is a pyrethroid. This may be a contributory factor to the build-up of insecticide resistance in local mosquito populations. The continuous exposure adds up to mosquitoes becoming strongly resistant to pyrethroids as well as other commonly used insecticides [49].

In Ghana organophosphates and carbamates are used interchangeably in IRS and this may have contributed to an extent the high resistance of *Anopheles gambiae* (*s.l*.) to these insecticides. High resistance to carbamates was observed notwithstanding cross-resistance with organophosphates, to which *Anopheles gambiae* (*s.l*.) were highly susceptible. This was as a result of reported complexity of insecticide resistance mechanism in mosquitoes of which Pedra et al. [50] indicated that wider range of genes are likely to be involved than the already stipulated genes. In addition to the target site-insensitivity as a result of point mutation or structural modification, increased metabolic detoxification of insecticides in mosquitoes is the most implicated mechanism [51]. Hence resistance observed to carbamates could be a result of metabolic detoxification of this insecticide by *Anopheles* spp. There is another school of thought that suggests that carbamates in contrast to organophosphates have reversible action on cholinesterase. The enzymatic activity is restored within a short while as carbamates lose their potential to restrain anticholinesterase thus rendering them less effective [52].

Effective insecticide resistance management is highly essential in preventing resistance, regain susceptibility or delay the development of resistance in mosquitoes to support and improve public health. An all-inclusive integrated vector control strategy including biological, chemical, physical, molecular, environmental and educational aspects targeting the various stages of the vectors, is long overdue and should be implemented in all malaria endemic countries as soon as possible. Ghana as a nation has adapted an insecticide resistance management plan, which was informed based on a nationwide survey, conducted to identify gaps in insecticide resistance data in the country. The plan comprises of the setting up of sentinel sites for the routine monitoring of insecticide resistance by renowned research institutions in the country. This exercise is thus ongoing throughout the nation, which will inform the next stage of action to be taken [45].

Our study had some limitations. Even though sampling was from the most populous region of Kumasi, mosquito larvae were from farms and not households. It is possible that the sampled population may not reflect exactly that which pertains all over Ashanti region. Therefore one ought to interpret these results with caution. That notwithstanding, several studies have revealed similar *Anopheles* population comparable to findings in this study.

## Conclusions

Since the high use of insecticides in irrigated farms in urban areas are probably a contributory factor to the development of resistance in the malaria vector, there should be continuous monitoring of use. The high coverage of LLINs and other insecticide malaria control measures in urban areas may strongly select for *kdr* mutations hence the high prevalence in *An coluzzii* (91%) and *An. gambiae* (*s.s*.) (61%). This study reveals that *An. gambiae* (*s.l*.) from Asokwa submetropolis in the Ashanti Region are resistant to all classes of insecticides except for organophosphates. Pirimiphose-methyl (0.25%) belonging to the organophosphates is the most effective insecticide against malaria vectors. Pyrethroids, the most widely used insecticide in ITNs and IRS had the highest level of resistance in the population of mosquito vectors. Our study therefore advocates the use of organophosphates in IRS as a malaria control tool boost to LLINs in these areas while effective insecticide resistance management plan is adapted and strictly adhered to.

## Abbreviations

*ace*-*1*: Acetylcholinesterase
AS: Allele-specific
CHRPE: Committee on human research publication and ethics
CTAB: Cetyl trimethyl ammonium bromide
DDT: Dichlorodiphenyltrichloroethane
IRS: Indoor residual spraying
ITN: Insecticide-treated nets
KCCR: Kumasi centre for collaborative research into tropical medicine
KD: Knockdown effect
*kdr*: knockdown resistance
KNUST: Kwame Nkrumah University of Science and Technology
LLINs: Long-lasting insecticide nets
PCR: Polymerase chain reaction
WHO: World Health Organisation
